# Novel combined variants of *WT1* and *TET2* in a refractory and recurrent AML patient

**DOI:** 10.1186/s12920-021-01002-0

**Published:** 2021-06-13

**Authors:** Qiang Ma, Yixian Guo, Xiaoxi Lan, Guoxiang Wang, Wanling Sun

**Affiliations:** Department of Hematology, Xuanwu Hospital, Capital Medical University, No. 45 Changchun Street, Beijing, 100053 People’s Republic of China

**Keywords:** Acute myeloid leukemia, *WT1*, *TET2*, Germline variant, Next-generation sequencing, Prognosis, Case report

## Abstract

**Background:**

Somatic mutations in Wilms' tumor 1 (*WT1*) and tet methylcytosine dioxygenase 2 (*TET2*) genes were separately perceived as contributors to hematopoietic disorders and usually thought to have a mutually exclusive effect in acute myeloid leukemia (AML). However, we found novel *WT1* and *TET2* variants persistently co-existed in a refractory and recurrent AML patient with t(9;11)(p21.3;q23.3); *KMT2A-MLLT3*, and were only detectable genetic alteration in early recurrence. Hence, these two novel variants were further investigated in patient’s family, and the potential effect on disease progression was evaluated at follow-up.

**Case presentation:**

A 27-year-old male was diagnosed with AML, having t(9;11)(p21.3;q23.3); *KMT2A-MLLT3*, accompanied by *WT1* (NM_024426.6:exon7:c.1109G>C:p.Arg370Pro) and *TET2* (NM_001127208.3:exon11:c.5530G>A:p.Asp1844Asn) variants. After two cycles of induction chemotherapy, complete remission was achieved. A consolidation treatment was then completed. However, the evaluation of the bone marrow revealed that early recurrence, *WT1* (p.Arg370Pro) and *TET2* (p.Asp1844Asn) variants still detectable, instead of *KMT2A-MLLT3*. Subsequently, these two variants were proved to be germline variants, which inherited from father and mother respectively. And the patient's elder brother also carried *TET2* (p.Asp1844Asn) variant. A sequential allogeneic HLA-matched sible hematopoietic stem cell transplantation (allo-HSCT) was carried out, and the donor is the patient's elder brother, the original two variants of patient were replaced by the donor-derived *TET2* (p.Asp1844Asn) variant after allo-HSCT; the patient has remained in complete remission with regular follow-up.

**Conclusions:**

In brief, it is firstly reported that *WT1* p.Arg370Pro and *TET2* p.Asp1844Asn variants co-existed in a refractory and recurrent AML patient by inheritance. These two variants of the patient were replaced with donor-derived *TET2* p.Asp1844Asn after allo-HSCT, and the patient has remained in complete remission with regular follow-up.

## Background

Acute myeloid leukemia (AML) is a clonal hematopoietic disorder and is usually observed along with genetic alterations in hematopoietic stem cells [1]. Most of the molecular cytogenetic abnormalities involved in AML have been identified, and these may be potential biomarkers for differential diagnosis, risk stratification, and therapeutic response in AML patients [2, 3]. Nevertheless, genetic alterations with definite clinical significance are relatively rare [4], and the clinical significance of most of them is still unknown [5], especially for germline mutations [6].

In our hospital, a 27-year-old male was diagnosed with AML, having t(9;11)(p21.3;q23.3); *KMT2A-MLLT3*, accompanied by Wilms' tumor 1 (*WT1*, NM_024426.6:exon7:c.1109G>C:p.Arg370Pro) and tet methylcytosine dioxygenase 2 (*TET2*, NM_001127208.3:exon11:c.5530G>A:p.Asp1844Asn) variants, without abnormal *WT1* mRNA expression. However, there are some noteworthy issues in this case, for instance: Why did *WT1* and *TET2* variants, which were previously reported to be mutually exclusive [7, 8], persistently co-exist in the AML patient? *WT1* (p.Arg370Pro) and *TET2* (p.Asp1844Asn) variants were only detectable genetic alterations in patients with early recurrence, instead of *KMT2A-MLLT3*, and whether co-exist of these two variants contribute to disease progression? To address these issues, blood and buccal mucosa cells samples of patient and his family were firstly collected, targeted multi-genes panel sequencing was performed to determine whether variation is hereditary or acquired. And after allo-HSCT, targeted multi-genes panel sequencing was performed again to investigate whether the variants were replaced, and regularly follow-up was performed.

## Case presentation

**Fig. 1:**
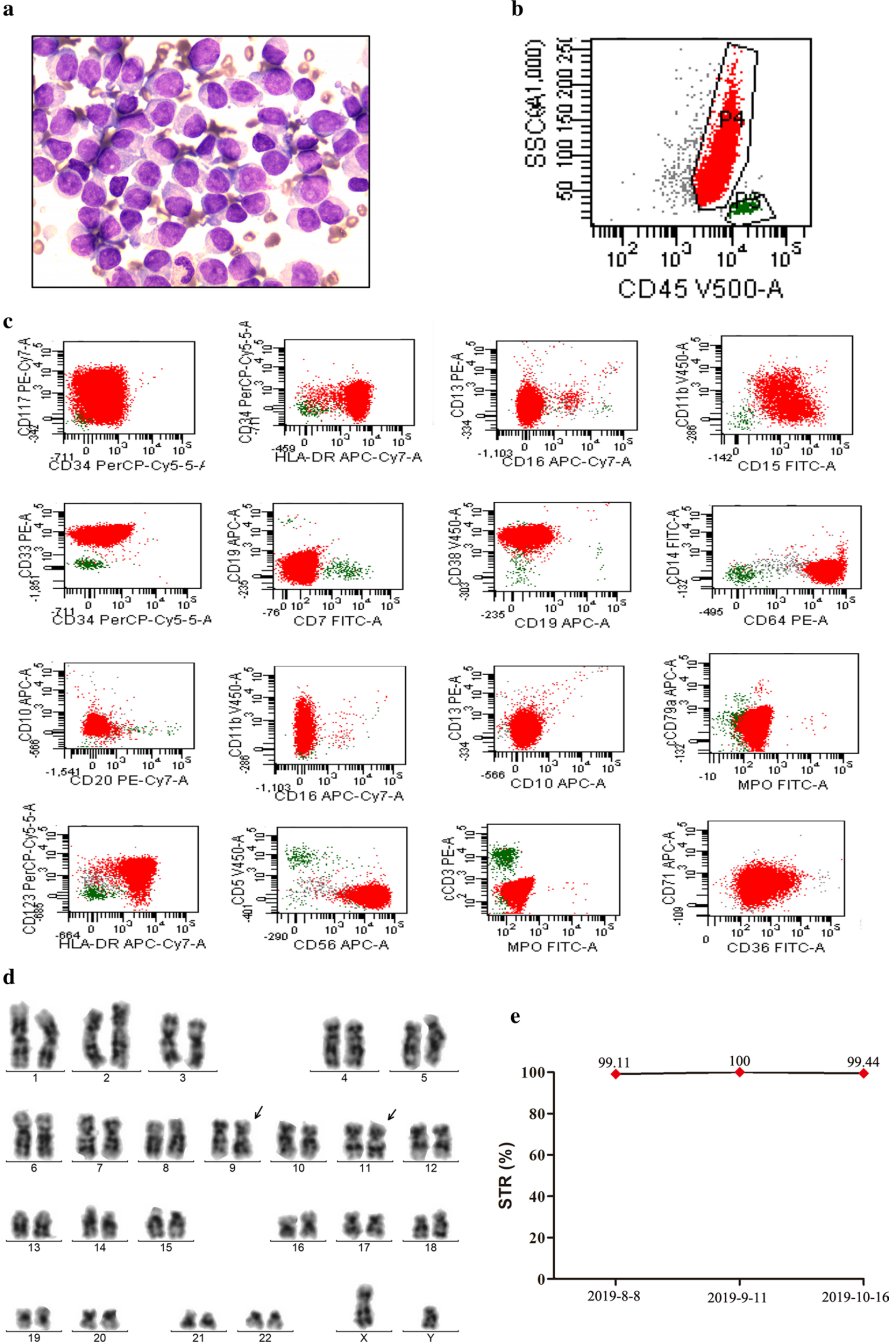
Representative results of laboratory tests of the patient with AML. **a** Monoblasts were observed in bone marrow of the patient, and some had very abundant cytoplasm(OLYMPUS BX53; Smart Digital Camrea;original magnification, ×1000); **b**, **c** P4 stands for abnormal cells, accounted for 96%. HLA-DR(+), CD33(+), CD15(+), CD64(+), CD38(+), CD123(partly+), CD56(+);CD11b(partly +), CD34(partly+), CD117(partly+); CD10(−), CD19(−), CD20(−), MPO(−), cCD3(−), cCD79a(−), CD13(−), CD7(−), CD5(−), CD14(−). **d** Chromosome karyotype analysis showed that the patient with 46, XY, t(9;11)(p21.3;q23.3). **e** Complete donor chimerism was observed after allo-HSCT by short tandem repeat polymerase chain reaction (STR-PCR) analysis

A 27-year-old male was admitted to our hospital in Jan 2019 due to hypoleukocytosis lasting two weeks. His family history did not suggest an inherited susceptibility to cancer, as no first-degree relatives, including his parents, his brother, and his child, had cancer. The complete blood cell count (CBC) indicated a white blood cell (WBC) count of 0.77 × 10^9^/L, platelet count of 129 × 10^9^/L, and hemoglobin (Hb) concentration of 45 g/L. Bone marrow aspiration revealed monocyte hyperplasia with 93% monoblasts and 2.5% promonocytes (Fig. 1a). Moreover, non-specific esterase stain revealed intensely positive monoblasts, and the positive stain could be inhibited by sodium fluoride (data not shown). Immunophenotype analysis of the abnormal cells by flow cytometry revealed full expression of HLA-DR, CD38, CD33, CD15, CD64 and CD56; partial expression of CD34, CD117, CD123, CD11b; and no expression of CD13, CD7, CD5, CD19, CD10, CD20, CD14, MPO, cCD79a and cCD3 (Fig. 1b, c). Chromosome analysis of bone marrow cells indicated a karyotype of 46, XY, t(9;11)(p21.3;q23.3) (Fig. 1d). The *KMT2A-MLLT3* fusion gene was identified using qRT-PCR.Targeted multi-genes panel sequencing revealed that *WT1* (p.Arg370Pro, VAF 49.19%) and *TET2* (p.Asp1844Asn, VAF 25.25%) variants were present. He was diagnosed with AML, having t(9;11)(p21.3;q23.3), *KMT2A-MLLT3*, accompanied by *WT1* and *TET2* variants, intermediate risk. A standard IA induction chemotherapy regimen (idarubicin + cytarabine) was administered from January 30, 2019.

At the end of first induction chemotherapy on February 20, 2019, the CBC revealed a WBC count of 0.64 × 10^9^/L, Hb of 83 g/L, platelet count of 21 × 10^9^/L. Bone marrow aspiration showed that primitive and immature cells made up 6% of the nucleated bone marrow cells. Flow cytometry detected 21% abnormal immunophenotype monocytes. The patient achieved partial remission. The CAG (Clarithromycin + Cytarabine + G-CSF) regimen was then administered as the second induction chemotherapy from February 21, 2019.

The evaluation of the bone marrow on March 21, 2019, showed that primitive and immature monocytes made up 0% of the bone marrow nucleated cells. Monocytes with abnormal immunophenotype were less than 0.01%. The *KMT2A-MLLT3* fusion gene was not present. Complete remission was achieved. However, the *WT1* (p.Arg370Pro VAF 50.11%) and *TET2* (p.Asp1844Asn VAF 48.33%) variants were still present. Another IA regimen was then administered as consolidation chemotherapy from March 29, 2019.

The evaluation of the bone marrow on May 9, 2019 revealed that primitive and immature monocytes made up 7% of the nucleated bone marrow cells and CD34+ CD117+ CD33+ cells were 0.75% by flow cytometry. *KMT2A-MLLT3* fusion remained negative. All these data suggested early recurrence. While *WT1* (p.Arg370Pro) and *TET2* (p.Asp1844Asn) variants were still positive, the VAF was 51.87% and 51.78%, respectively.

Owing to the fluctuation of the disease, the risk stratification was re-evaluated to high-risk. A HA regimen chemotherapy (homoharringtonine and intermediate-dose cytarabine) was administered from May 11, 2019. The second complete remission was achieved, with variants on *WT1* (p.Arg370Pro) and *TET2* (p.Asp1844Asn) genes still present, and the VAF was 48.35% and 49.88%, respectively (Table 1).

**Table 1:** Molecular and cellular genetic alternation of patient at different disease stages

Examination	Before allo-HSCT				After allo-HSCT		
	Initial diagnosis	CR1	Relapse	CR2	1	2	3
Karyotype	t (9; 11) (p21.3;q23.3)	NA	–	NA	–	–	NA
Fusion	*KMT2A-MLLT3*	–	–	NA	–	NA	NA
Variants (VAF %)	*TET2* (25.25)	*TET2* (48.33)	*TET2* (51.78)	*TET2* (49.88)	*TET2* (48.58)	*TET2* (50.20)	*TET2* (52.59)
	*WT1* (49.19)	*WT1* (50.11)	*WT1* (51.87)	*WT1* (48.35)			
*WT1* level (%)	0	NA	0.03	0.15	NA	NA	0

– Normal karyotype or fusion gene negative; *allo-HSCT* Allogeneic hematopoietic stem cell transplantation, *NA* Not available, *CR1* complete remission after two cylces inductive chemotherapy, *CR2* complete remission after one cycle reinductive chemotherapy; WT1 level (%) = (WT1 mRNA copies/ABL1 mRNA copies) × 100, reference range of WT1 mRNA level in our laboratory is 0–0.6%. WT1:NM_024426.6:exon7:c.1109G>C:p.Arg370Pro; TET2:NM_001127208.3:exon11:c.5530G>A:p.Asp1844Asn

A sequential allogeneic HLA-matched sible hematopoietic stem cell transplantation was carried out on July 9, 2019, and the donor is patient’s elder brother. After allo-HSCT, complete donor chimerism was received (Fig. 1e), and donor-derived *TET2* p.Asp1844Asn variant was also received. To date, the patient has remained in complete remission with regular follow-up.

Since the patient's variants were persistent and does not change with treatment, we suspected the *WT1* (p.Arg370Pro) and *TET2* (p.Asp1844Asn) variants were germline variants. Then buccal mucosa cells (somatic control) were first obtained from the patient for targeted sequencing, and results revealed that shared variation sites of *WT1* p.Arg370Pro (52.09%) and *TET2* p.Asp1844Asn (37.26%) existed in buccal mucosa cells, indicating that *WT1* p.Arg370Pro and *TET2* p.Asp1844Asn may germline variants.

**Fig. 2:**
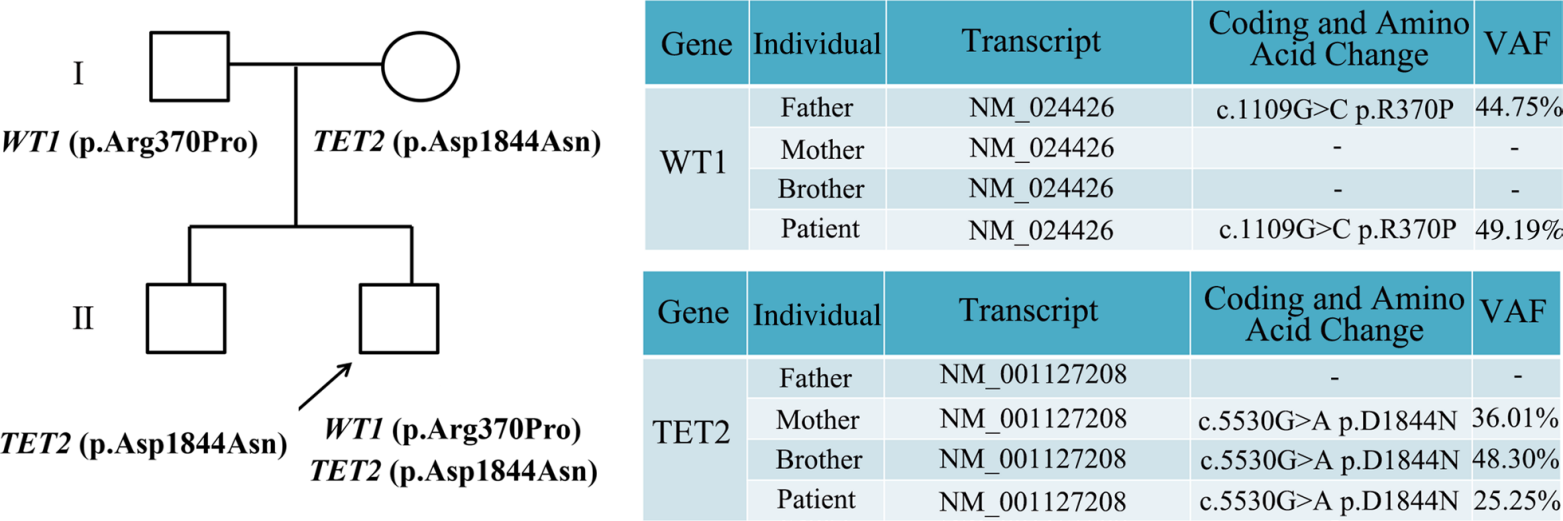
Genetic change of *WT1* and *TET2* in patient’s relatives. Variants status and VAF value of *WT1*/*TET2* in the patient’s relatives

To further identify whether these two variants were heritable, DNA samples from the patient’s relatives were analyzed using the same target sequencing; *WT1* p.Arg370Pro (VAF 44.75%) was detected in the patient’s father and *TET2* p.Asp1844Asn (VAF 36.01%) was detected in his mother; the brother carried the *TET2* p.Asp1844Asn variant (VAF 48.30%) (Fig. 2). The above results thoroughly confirmed that germline variants *WT1* p.Arg370Pro and *TET2* p.Asp1844Asn co-existed in the patient by inheritance.

## Discussion and conclusions

In this study, we first reported that *WT1* p.Arg370Pro co-exists with *TET2* p.Asp1844Asn variant in a refractory and recurrent AML patient. These two variants were derived from the patient’s father and mother respectively, and were only detectable genetic alteration in early recurrence. These two variants were replaced with donor-derived *TET2* p.Asp1844Asn variant after allo-HSCT, and the patient has remained in complete remission with regular follow-up.

*WT1* and *TET2* mutations were mutually exclusive in AML patient, it is rare that *WT1* and *TET2* variants co-exist in an AML patient [8]. As the co-existing variants in this case were germline variants derived from the patient’s father and mother, respectively, this case serves as a reminder that mutually exclusive germline variants may co-exist in a patient due to inheritance. Recently, a growing number of germline mutations associated with tumor susceptibility have been reported; for instance, *MITF* (Microphthalmia-associated transcription factor) mutation impairs SUMOylation, which has an oncogenic function in the tumorigenesis of multiple tissues/melanocytes and kidney cells [9]. *GATA2*, *RUNX1*, *DDX41*, *PAX5*, *CEBPA*, and *TP53* are associated with hematologic malignancies predisposition [10]. Identifying germline mutations is beneficial for therapy choice, donor selection for hematopoietic stem cell transplantation, evaluation of comorbidities, and surveillance strategies to improve the clinical outcomes [10].

Although it is unclear whether co-exist of these two variants closely associated with AML predisposition and recurrence, *WT1* and *TET2* genes are necessary for the normal development of blood cells based on previous findings. *TET2* is important for normal myelopoiesis, and disruption of *TET2* enzymatic activity favors myeloid tumorigenesis [11]. Slower proliferation and reduced clonogenicity have been observed in *TET2* overexpressing cells in vitro compared to those transfected with the empty vector, which indicates the suppressor role of *TET2* in AML. However, the inhibitory effects of *TET2* on leukemic cell proliferation and colony formation could be abolished by *WT1* knockdown [11]. Further studies revealed that *TET2* suppresses leukemia cell proliferation and colony formation in a *WT1-*dependent manner [12]. All of the above conclusions are from in vitro and animal experiments; here, for the first time, *TET2* and *WT1* variants are reported to co-exist in an AML patient, with detectable alterations only in the complete remission and relapse stages. Interesting, when these two variants were replaced with donor-derived *TET2* p.Asp1844Asn variant, patient has been received complete remission during follow-up. It is necessary to explored whether co-exist of *WT1* and *TET2* variants have a worse effect on disease progression compared with existed alone.

Since the interaction of these two genes is essential for biological functions, we attempted to explain the pathogenesis at the molecular level. *TET2* binds to the zinc finger domain (residue 323–449), but not the N-terminal region (residue 1–323) of *WT1*. In this patient, the missense mutation was in the zinc finger domain (residue 370) of *WT1*, which may disrupt the binding between *WT1* and *TET2.* Moreover, *WT1* binds to the CD domain of *TET2*, but not the N-terminal region (residue 1 to 1127) [11]. Unfortunately, in this case, the missense mutation was in the CD domain (residue 1844) of *TET2*, which may further disrupt the binding between *WT1* and *TET2*. From a molecular point of view, this may be a reasonable explanation for the disease. In clinical practice, *WT1* expression and disease progression are usually parallel to each other; *WT1* expression is also used as the MRD marker of hematological neoplasms when other genetic alterations are absent [13]. However, in this case, abnormal up-regulation of *WT1* was not observed at different stages, but the *WT1* variant persisted. Similar to the *WT1* mutation being related to poor prognosis [14], *WT1* overexpression was also indicated to be associated with shorter OS [15]. However, the question remains, which alteration could be a potential indicator of prognostic stratification for the patient; expression level or mutation status? It seems contradictory for the patient to have *WT1* variant and no *WT1* overexpression, and related reports in the literature are limited.

In this case, *WT1* and *TET2* variants were the only molecular alterations in the relapse stage. Then, this is only a phenomenon we observed, and the existing evidence is not enough to show that co-existing *WT1* and *TET2* variants were involved in the occurrence and progression of AML in the above manner. Previous studies have revealed that *WT1* can bind *TET2*, acting as a guide for *TET2* to specific genomic loci, leading to increased stem cell function and self-renewal with proliferation [16]. This seems a reasonable explanation for why the patient relapsed in a short time, but other possible reasons also need to be further explored. However, after allo-HSCT, two variants of patient were replaced with donor-derived *TET2* p.Asp1844Asn, then patient has remained in complete remission with regular follow-up, we have to speculate whether there was the possibility of concurrent presence of *WT1* gene mutation could contribute to second hit in the pathogenesis of AML. In brief, these two variants might play some role in the pathogenesis of refractory and recurrent AML, while a long term follow-up and regular monitoring by NGS is needed. At the same time, we just report a case and the result of the family pedigree study. More cases and related families, and further researches are necessary to prove our hypothesis.

In summary, we present the first report that *WT1* and *TET2* variants co-existed in a refractory and recurrent AML patient; these were identified as germline variants. After allo-HSCT, the original variants were replaced by the donor-derived *TET2* variant; the patient has remained in complete remission with regular follow-up.

## Data Availability

The patient’s DNA sequencing data generated during the current study are deposited in the NCBI Sequence Read Archive (SRA) repository under the accession number PRJNA729676. The datasets generated and/or analyzed during the current study are available in the Genbank repository (GRCh37/hg19, https://www.ncbi.nlm.nih.gov/genome/guide/human/) for NM_024426.6 (https://www.ncbi.nlm.nih.gov/nuccore/NM_024426.6/) and NM_001127208.3 (https://www.ncbi.nlm.nih.gov/nuccore/NM_001127208.3). The raw datasets of family members generated during the current study are not publicly available because it is possible that individual privacy could be compromised. It is possible to apply for permission to obtain access to the raw sequencing data through the corresponding author.

## Abbreviations

AML: Acute myeloid leukemia
TET2: Tet methylcytosine dioxygenase 2
WT1: Wilms' tumor 1
MRD: Minimal residual disease
VAF: Variant allele frequency
CBC: Complete blood cell count
WBC: White blood cell count
Hb: Hemoglobin
HSCT: Hematopoietic stem cell transplantation
qRT-PCR: Quantitative real-time PCR
OS: Overall survival
